# A Case of Superficial Acral Fibromyxoma of the Index Finger

**DOI:** 10.7759/cureus.60518

**Published:** 2024-05-17

**Authors:** Samantha A Riebesell, Johnlevi S Lazaro, David Kirby, Michael Rivlin

**Affiliations:** 1 Hand Surgery, Rothman Orthopedic Institute, Philadelphia, USA; 2 Orthopedic Surgery, Jefferson Health New Jersey, Stratford, USA

**Keywords:** benign, digit, digital fibromyxoma, acral soft tissue tumor, superficial acral fibromyxoma

## Abstract

Superficial acral fibromyxoma (SAFM) is a rare, slow-growing benign soft tissue tumor that is typically asymptomatic in nature and usually affects the acral regions of the hands and feet. The majority of these lesions are subungual. Excisional biopsy is the primary treatment modality. Despite the distinct clinical and histopathological features, misidentification of this slow-growing tumor persists. This case report contributes to the existing literature by delineating the clinicopathologic features, radiographic and MRI findings, and treatment strategies of SAFM.

## Introduction

Superficial acral fibromyxoma (SAFM), also known as digital fibromyxoma, is a rare, benign, slow-growing soft tissue tumor primarily affecting the acral regions of the hands and feet. While typically asymptomatic, SAFM can cause significant pain due to associated nail deformities as it progresses [1]. First documented in a case series by Fetsch et al. in 2001, SAFM demonstrates a male predominance, manifesting as a solitary mass ranging from 0.6 to 5.0 cm in size in the subungual region [2]. Cullen et al. have separately reported SAFM on the sole of the foot and the wrist in addition to the digits [3]. Histopathologically, SAFMs feature moderately cellular and contain spindled and stellate-shaped fibroblast-like cells within a myxoid or myxocollagenous matrix that contain increased vasculature and mast cells. SAFM presents as a non-encapsulated soft tissue tumor of the dermis and appears as loosely storiform growth patterns [3]. Nuclear atypia if present is typically of low degree and mitotic figures are uncommon [2,4]. On immunohistochemical staining, SAFM typically stains positive for CD34, CD99, and Vimentin, with scant positivity for S100 protein [2,5]. Fetsch et al. reported positive staining for CD34, epithelial membrane antigen (EMA), and CD99, and no immunoreactivity for actins, desmin, keratins, or HMB-45 [2].

The primary treatment modality for SAFM involves surgical excision to mitigate the risk of recurrence [6]. Despite the extensive literature on SAFM, misidentification remains a challenge, emphasizing the need for continued elucidation of its clinicopathologic features, imaging characteristics, and treatment modalities. This case report contributes to the existing literature by presenting a comprehensive evaluation of SAFM involving the index finger, encompassing clinical findings, radiographic and MRI observations, and the surgical management approach.

## Case presentation

**Figure 1:**
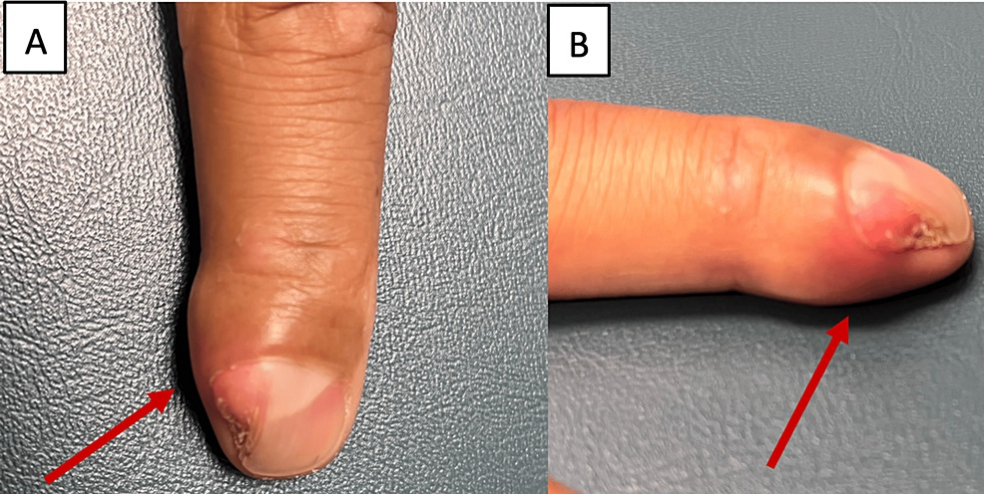
Clinical photographs of lesion. (A) Dorsal view. (B) Radial view.

A 45-year-old right-hand dominant male who works in Information Technology presented to the office with a right index finger deformity and nail deformity of the distal phalanx that he had noticed three years back. The patient reported pain and progressive growth of the lesion with no antecedent injury or trauma. He also denied any numbness or tingling in the affected area. Upon physical exam, the right index finger demonstrated a palpable mass and nail plate deformity (Figures 1A, 1B). Sensation and capillary refill were within normal limits.

**Figure 2:**
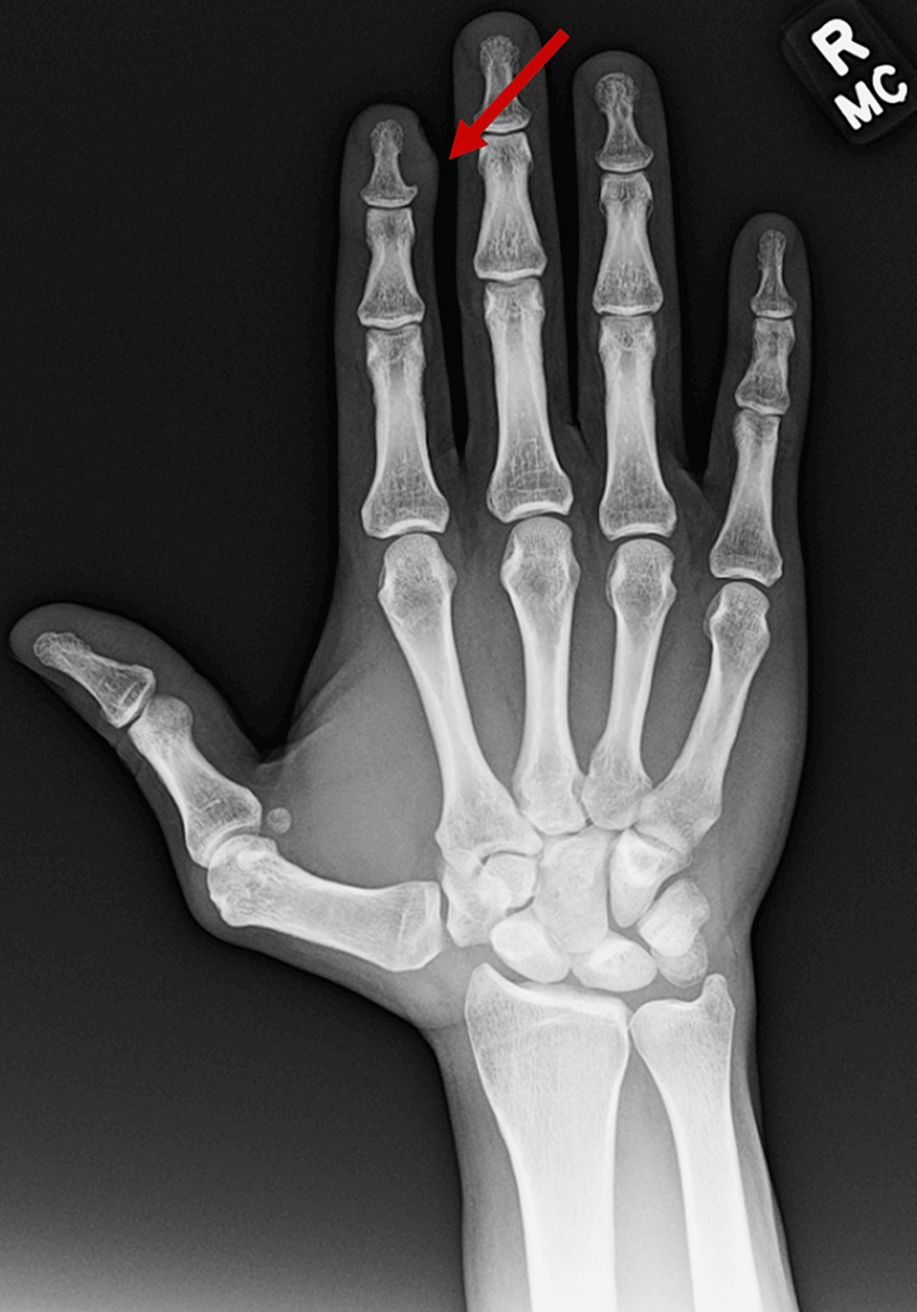
Plain radiograph of the right hand.

**Figure 3:**
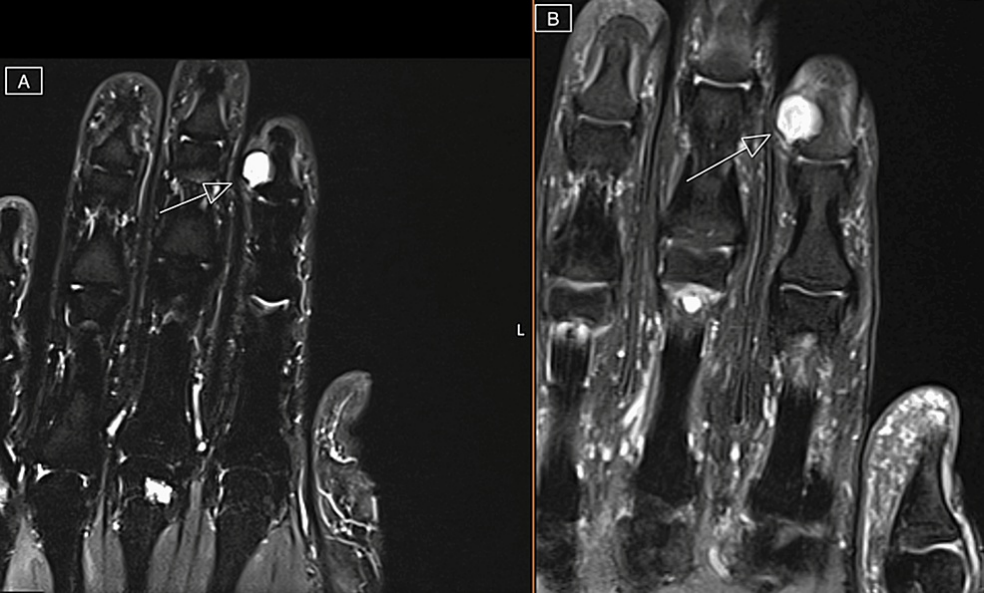
Coronal view of initial MRI scan depicting a parosteal lesion, likely to be a glomus tumor of the right index finger. (A) Post-contrast. (B) T2-weighted fat suppressed.

**Figure 4:**
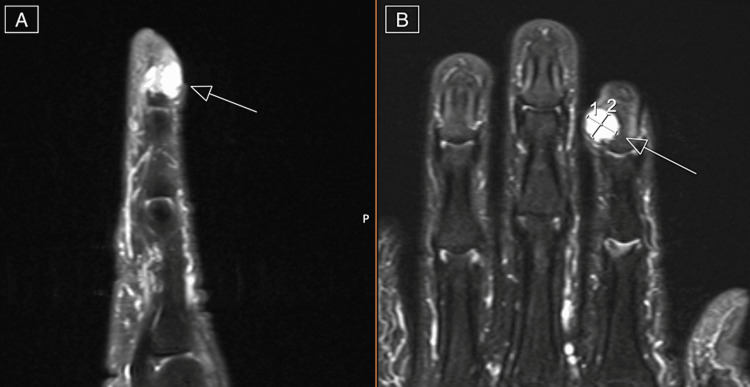
T2-weighted fat-suppressed images of MRI revealing hyperintense possible ostial lesion of the distal phalanx. (A) Sagittal view. (B) Coronal view.

Initial plain radiograph imaging demonstrated a soft tissue shadow over the dorsal distal phalanx of the index finger with slight asymmetry of the bone cortex without erosion (Figure 2). A year prior to the current presentation the patient was seen by an outside provider who obtained an MRI. This initial MRI revealed a parosteal lesion, which was interpreted by radiology to be a glomus tumor of the right index finger (Figures 3A, 3B). Given the lapse in timing from the initial MRI, a repeat MRI was performed which demonstrated a stable, nonaggressive appearing T2 hyperintense mass of the distal phalanx which measured to be 8 mm x 11 mm x 10 mm with erosion of the bone (Figures 4A, 4B). Based on the physical exam and radiographic and MRI findings, surgery was recommended.

**Figure 5:**
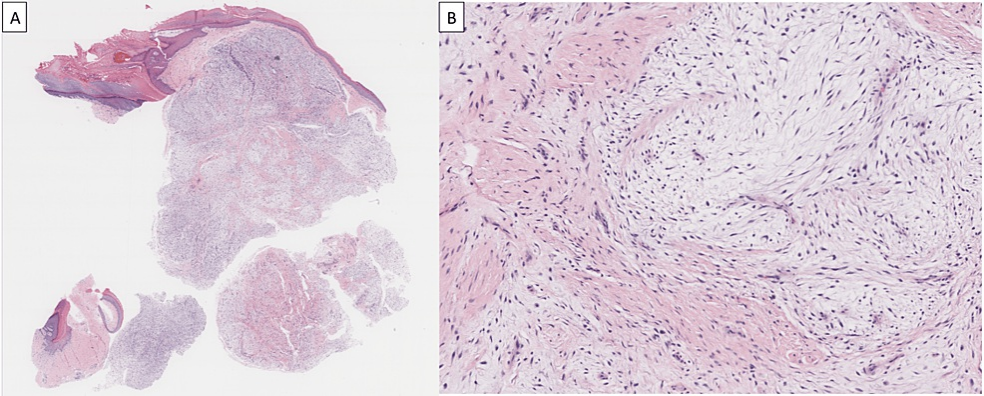
Histochemical hematoxylin and eosin (H&E) staining. (A) Acral skin lesion. (B) Fibromyxoid stroma composed of spindles to stellate cells in a loose storiform pattern.

**Figure 6:**
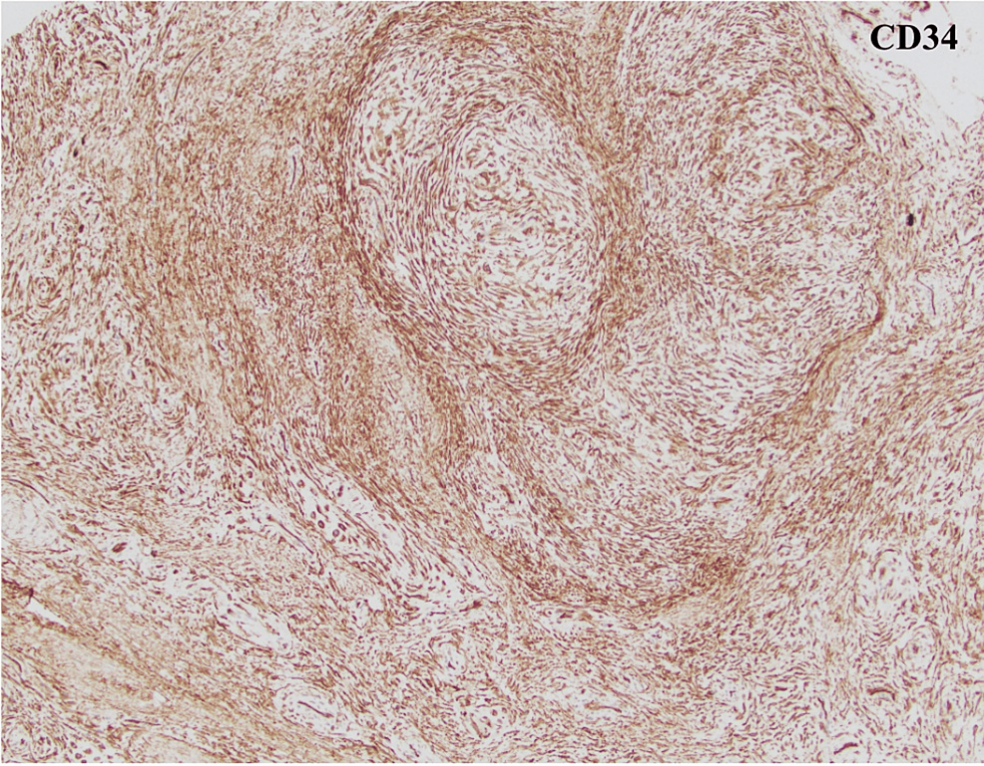
On immunohistochemical staining, cells were diffusely positive for CD34.

**Figure 7:**
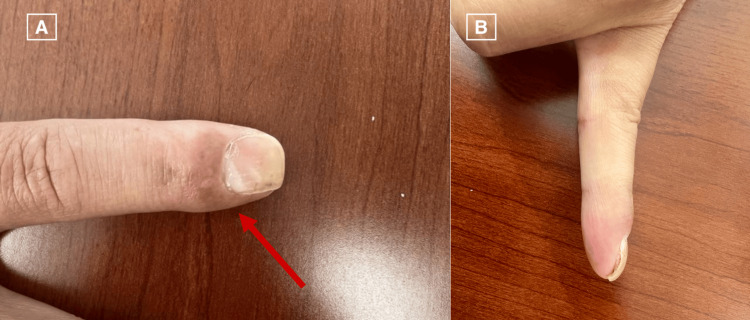
Clinical photograph from the third postoperative visit (two months following surgery). (A) Dorsal view. (B) Radial view.

**Figure 8:**
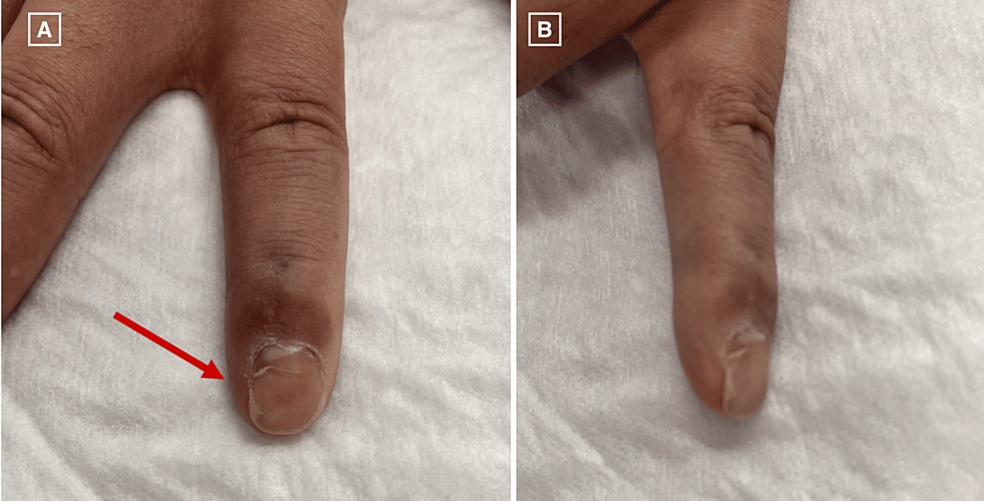
Clinical photograph from the fourth postoperative visit (four months following surgery). (A) Dorsal view. (B) Radial view.

Surgical mass excision with nail plate removal and bed repair was performed. Monitored Anesthesia Care (MAC) sedation with a digital block was performed. The nail plate was removed, and the edge of the tumor was identified. The sterile matrix of the nail bed was sharply incised, and a marginal excision of the tumor was performed. The tumor was found to involve the distal phalanx periosteum; however, the tumor was removed without affecting the integrity of the distal phalanx. The sterile matrix was repaired with 5-0 absorbable gut suture and the nail fold was splinted. The specimen was sent for histopathological examination (Figures 5A, 5B), which demonstrated fibromyxoid stroma composed of spindled to stellate cells in a loose storiform or fascicular growth pattern. Mast cells were easily identifiable and lesional cells showed only mild nuclear atypia with no nuclear pleomorphism or mitotic activity. On immunohistochemical staining, cells were diffusely positive for CD34 (Figure 6) and vimentin and negative for S100, SMA, desmin, and cytokeratin. Following the procedure, the patient was seen for three postoperative visits and initially was told to keep his hand in the splint and begin hand therapy. At almost two months postoperatively, the patient’s pain and range of motion improved without any signs of infection (Figures 7A, 7B). The most recent postoperative visit at approximately four months from surgery showed reduced swelling with the wound completely healed and improved range of motion (Figures 8A, 8B). The patient’s pain had subsided, and the patient was instructed to resume activities as tolerated.

## Discussion

This case of SAFM underscores the diagnostic complexities and treatment considerations inherent in managing this rare soft tissue tumor. The patient’s clinical presentation, characterized by a progressive deformity of the right index finger over three years, aligns with the indolent nature of SAFM. Notably, the absence of trauma or injury underscores the insidious progression of the tumor, often masked by its asymptomatic nature until significant deformity ensues. Despite the substantial nail deformity, the patient maintained a full range of motion and intact neurovascular status, reflecting SAFM's propensity for causing localized structural changes without compromising digit function, as reported in previous studies [3,5-8].

The initial radiographic evaluation revealed nonspecific soft tissue shadow and subtle bone cortex asymmetry, warranting further imaging. An MRI provided more information in characterizing the lesion as a parosteal mass with T2 hyperintensity, suggesting a possible glomus tumor. A subsequent MRI performed more than a year after the initial MRI evaluation revealed a stable, nonaggressive lesion with erosion of the distal phalanx. Advanced imaging provided insight into potential diagnoses allowing us to move forward with surgical excision for symptomatic treatment and histopathological examination of the lesion.

Mass excision with nail plate removal and nail bed repair/reconstruction was chosen to reduce the risk of recurrence [1,2,7,9-11]. The decision to perform surgery was supported by the clinical presentation and imaging. The histopathological examination of the excised specimen confirmed the diagnosis of SAFM, revealing a superficial dermal and subcutaneous lesion with characteristic fibromyxoid stroma in a storiform pattern which was further supported by positive staining for CD34 and vimentin, consistent with previous reports [2,5]. Postoperatively, the patient demonstrated improvement in pain and no signs of recurrence at approximately four-month follow-up. This positive outcome aligns with the general success of surgical excision as the primary treatment modality for SAFM.

## Conclusions

SAFM is a rare soft tissue tumor with distinctive clinical and histopathological features. This case report highlights the importance of recognizing SAFM in the differential diagnosis of acral masses. Increased awareness of this soft tissue tumor, combined with accurate histopathological examination and appropriate advanced imaging, is crucial for the successful diagnosis and appropriate management of SAFM. Further research and accumulation of cases are necessary to refine diagnostic criteria and establish optimal treatment strategies for this uncommon soft tissue tumor.
